# TMEM59 Haploinsufficiency Ameliorates the Pathology and Cognitive Impairment in the 5xFAD Mouse Model of Alzheimer’s Disease

**DOI:** 10.3389/fcell.2020.596030

**Published:** 2020-10-28

**Authors:** Jian Meng, Linkun Han, Naizhen Zheng, Hui Xu, Zhaoji Liu, Xian Zhang, Hong Luo, Dan Can, Hao Sun, Huaxi Xu, Yun-wu Zhang

**Affiliations:** ^1^Fujian Provincial Key Laboratory of Neurodegenerative Disease and Aging Research, Institute of Neuroscience, School of Medicine, Xiamen University, Xiamen, China; ^2^Department of Neurology, Zhongshan Hospital Xiamen University, Xiamen, China; ^3^Department of Neurology, The First Affiliated Hospital of Xiamen University, Xiamen, China

**Keywords:** Alzheimer’s disease, amyloid-β, cognitive deficits, neurite dystrophy, synaptic plasticity, TMEM59

## Abstract

Alzheimer’s disease (AD) is a progressive neurodegenerative disease associated with cognitive deficits and synaptic impairments. Amyloid-β (Aβ) plaque deposition, dystrophic neurite accumulation and neurofibrillary tangles are pathological hallmarks of AD. TMEM59 has been implicated to play a role in AD pathogenesis; however, the underlying mechanism remains unknown. Herein, we found that overexpression of TMEM59 in the hippocampal region led to memory impairment in wild type mice, suggesting its neurotoxic role. Interestingly, while TMEM59 overexpression had no effect on worsening synaptic defects and impaired memory in the 5xFAD mouse model of AD, it significantly exacerbated AD-like pathologies by increasing levels of detergent-insoluble Aβ and Aβ plaques, as well as dystrophic neurites. Importantly, haploinsufficiency of TMEM59 reduced insoluble Aβ levels, Aβ plaques, and neurite dystrophy, thereby rescuing synaptic plasticity and memory deficits in 5xFAD mice. Moreover, the level of TMEM59 in the brain of 5xFAD mice increased compared to wild type mice during aging, further corroborating its detrimental functions during neurodegeneration. Together, these results demonstrate a novel function of TMEM59 in AD pathogenesis and provide a potential therapeutic strategy by downregulating TMEM59.

## Introduction

Alzheimer’s disease (AD) is the most common age-related neurodegenerative disorder characterized by abnormal accumulation and deposition of various amyloid-β (Aβ) peptides derived from amyloid-β precursor protein (APP), formation of neurofibrillary tangles containing hyperphosphorylated tau, synaptic dysfunction, neuroinflammation, neuronal death, and cognitive decline (Crews and Masliah, 2010; Huang and Mucke, 2012; Guerreiro and Bras, 2015; Kocahan and Dogan, 2017; DeTure and Dickson, 2019). So far no effective therapies are available to cure this devastating disease (Yu et al., 2019). Since social and financial burdens for AD have become enormous as our population ages, there is an urgent need to elucidate the detailed molecular mechanisms underlying AD pathogenesis, so that new targets may be identified for therapeutic development.

Transmembrane protein 59 (TMEM59) (also known as dendritic cell-derived factor 1, DCF1) is a type I transmembrane glycoprotein ubiquitously expressed in various tissues. One study found that TMEM59 overexpression in cells induced APP retention in the Golgi, thereby inhibiting APP cleavage by α- and β-secretases at the plasma membrane and in the endosomes, respectively, resulting in reduced Aβ production (Ullrich et al., 2010). In addition, the *TMEM59* gene promoter region was found to be hypomethylated in postmortem frontal cortex of late-onset AD patients compared to controls; and methylation at this site was functionally associated with *TMEM59* mRNA and protein levels (Bakulski et al., 2012). Microarray data analysis also revealed that *TMEM59* gene expression was higher in AD patients than in controls (Guttula et al., 2012). Very recently another study reported that overexpression of TMEM59 reduced the cleavage of APP C99 fragment by γ-secretase and promoted learning and memory in drosophila expressing APP C99 (Li et al., 2020). All these studies suggest that TMEM59 is associated with AD. However, the exact role of TMEM59 in AD has yet to be fully determined.

In the current study, we explored the effects of TMEM59 on AD-associated phenotypes in mice and found that TMEM59 overexpression impaired memory in wild type (WT) mice and exacerbated Aβ and neurite pathologies in the 5xFAD mouse model of AD (Oakley et al., 2006). Importantly, TMEM59 haploinsufficiency rescued memory and synaptic plasticity deficits and reduced Aβ and neurite pathologies in 5xFAD mice. Moreover, we observed that TMEM59 expression was increased in the brain of 5xFAD mice.

## Materials and Methods

### Animals

*Tmem59* conditional knockout (*Tmem59^*floxflox*^*) mice were generated using a traditional homozygous recombination strategy with service provided by Cyagen Biosciences. Briefly, a homology region covering mouse *Tmem59* exon3 to exon6 was subcloned into the targeting vector. One Loxp site was introduced into *Tmem59* intron3, and another Loxp site together with a modified Rox-flanked Neo cassette was introduced into *Tmem59* intron5 (Supplementary Figure 1A). After linearization, the targeting vector was transfected into C57BL/6 background mouse embryonic stem cells. After G418 selection and confirmation of successful homologous recombination of the targeting vector, positive clones were injected into mouse blastocysts, which were then implanted into pseudo-pregnant females. Born chimeric mice (F0) were crossed with C57BL/6 mice to generate F1 mice carrying the recombined allele. The Neo cassette flanked by modified Rox sites was self-deleted during mouse production, with a confidential design by Cyagen Biosciences. *Tmem59*^+/–^ mice were generated by crossing *Tmem59*^*flox*/*flox*^ mice with Zp3-Cre mice (kindly provided by Haibin Wang) (Cheng et al., 2018).

The PCR primers used for genotyping were as follows:

*Tmem59*^flox/flox^, forward-5′-GAGTAGATGATGCTGACATA GAC-3′,
reverse-5′-CCTCTAAGGAGCTTTCTAAGTG-3′;
Zp3-Cre, forward-5′-CAGATGAGGTTTGAGGCCACAG-3′,
reverse-5′-TTCTTGCGAACCTCATCACTC-3′;
*Tmem59*^+/–^, WT-forward-5′-GAGTAGATGATGCTGACAT AGAC-3′,
KO-forward-5′-GTAAGAAACTAGAACTGGGCTTG AGC-3′,
reverse-5′-CCTCTAAGGAGCTTTCTAAGTG-3′.

5xFAD mice (Oakley et al., 2006) were crossed with *Tmem59*^+/–^ mice to generate 5xFAD; *Tmem59*^+/–^ mice. All mice were maintained and bred at Xiamen University Laboratory Animal Center. Mouse experimental procedures were performed in accordance with the National Institutes of Health Guide for the Care and Use of Laboratory Animals and approved by the Animal Ethics Committee of Xiamen University.

### Stereotactic Injection of Lentivirus

For TMEM59 overexpression in the hippocampus, WT and 5xFAD mice at 2 months of age were anesthetized, placed on a stereotaxic frame, and injected bilaterally with Lentivirus-EGFP control or Lentivirus-TMEM59-Flag (1 × 10^9^ v.g./mL, OBiO Technology) into the hippocampal region at the following coordinates: anterior posterior, −2.0 mm; medial lateral, ±1.5 mm; and dorsal ventral, −2.0 mm using an automated stereotaxic injection apparatus (RWD Life Science). Two μL lentivirus was delivered at 0.20 μL/min to each lateral. After each injection, the syringe was left for 10 min and then withdrawn slowly.

### Behavioral Tests

Mice at 6–7 months of age were subjected to behavioral tests including open field, *Y* maze and Morris water maze. Habituation was done in the testing room for more than 30 min at the beginning of each test day. All tests were carried out by researchers blinded to mouse genotype. Data were recorded and analyzed using Smart 3.0 video tracking system (Panlab, Harvard Apparatus).

For open field test (Tatem et al., 2014), each test mouse was placed in the center of the square box [40 cm (L) × 40 cm (W) × 40 cm (H)] and allowed to explore freely for 10 min. Time spent in the center and total distance of movement were measured.

For *Y* maze test (Miedel et al., 2017; Lachance et al., 2019), each test mouse was placed in the center of a “*Y*” shaped chamber [30 cm (L) × 6 cm (W) × 15 cm (H)] and allowed to enter into each arm freely for 5 min. The sequence of arm entries and total numbers of arms entered by each mouse was recorded. The percentage of alternation was calculated as the ratio of consecutive specific arm entries to the total arm entries.

The Morris water maze test (Vorhees and Williams, 2006; Bromley-Brits et al., 2011) was performed in a large circular pool (120 cm in diameter) filled with opaque water, in which a platform was hidden 1 cm below the water surface. Mice were subjected to two training trials per day for six consecutive days; and they were placed into the water facing the sidewalls of the pool from different start positions across trials. The time spent to reach and climb onto the platform (escape latency) was recorded. If a mouse failed to find the platform within 60 s, it was guided to the platform and allowed to stay on the platform for 10 s. On day 7, the platform was removed and a probe test for the mice was performed for 60 s. The time spent in each quadrant and the numbers of platform region crossings were recorded.

### Electrophysiology

LTP was recorded as previously described (Penn et al., 2017). Briefly, mice were anesthetized with isoflurane. After decapitation, the brain was rapidly transferred into an ice-cold solution (64 mM NaCl, 2.5 mM KCl, 10 mM glucose, 1.25 mM NaH_2_PO_4_, 10 mM MgSO_4_, 26 mM NaHCO_3_, 120 mM sucrose, and 0.5 mM CaCl_2_). The acute hippocampal slices (400 μm thick) were cut using a vibratome (VT1200S, Leica). Slices were allowed to recover for 1 h at 32°C and incubated for at least 1h at room temperature before recording in artificial cerebrospinal fluid (aCSF: 126 mM NaCl, 3.5 mM KCl, 1.25 mM NaH_2_PO_4_, 1.3 mM MgSO_4_, 2.5 mM CaCl_2_, 26 mM NaHCO_3_, and 10 mM glucose). All solutions were saturated with 95%O_2_/5%CO_2_ (volume/volume). fEPSPs were induced in the Schaffer collateral pathway with a two-concentrical bipolar stimulating electrode (FHC, Inc.). LTP was induced by two trains of stimuli at 100 Hz for 1 s with 30 s interval. fEPSP response was recorded for 1 h after tetanic stimulation. Data were acquired with Clampex 10.6 (Molecular Devices) and analyzed using Clampfit 10.6 software (Molecular Devices).

### Western Blotting

Mouse hippocampal and cortical tissues were homogenized and lysed in RIPA lysis buffer (25 mM Tris–HCl [pH 7.6], 150 mM NaCl, 0.1% SDS, 1% sodium deoxycholate, and 1% Non-idet P-40) supplemented with the Complete Protease and Phosphatase Inhibitor Cocktail (Roche). Protein concentrations were determined by a BCA Protein Assay Kit (Thermo Fisher Scientific) following the manufacturer’s instruction. 25 micrograms of total protein lysates were resolved using SDS-polyacrylamide gel electrophoresis and transferred to PVDF membranes. After blocking in 5% milk in 0.1% PBS/Tween-20, membranes were immunoblotted with indicated primary antibodies overnight at 4°C, and then incubated with appropriate horseradish peroxidase (HRP)-conjugated secondary antibodies for 1 h at room temperature. The antibodies used were: anti-TMEM59 (ABclonal, WG-03224D, 1:4000), anti-APP (22C11, Millipore, MAB348, 1:1000), anti-APP Carboxyl-terminus (369, 1:1000) (Xu et al., 1998), anti-Flag (Proteintech, 20543-1-AP, 1:1000), anti-α-tubulin (Millipore, MABT205, 1:5000), anti-β-actin (Cell Signaling Technology, 8457S, 1:2000), and HRP-conjugated secondary antibodies (Thermo Fisher Scientific, 31460 or 31430, 1:5000). Protein band intensity was quantified using the ImageJ software (National Institutes of Health).

### Immunostaining

Mice were anesthetized and intracardially perfused with ice-cold PBS and 4% paraformaldehyde. Brains were harvested and post-fixed for 2–4 h at 4°C. Brains were washed in PBS and cryoprotected in 30% sucrose in PBS. Coronal sections (15 μm thick) were collected with a freezing microtome (Leica). The sections were washed in PBS and then blocked in blocking buffer (5% BSA and 0.2% Triton X-100) for 1 h at room temperature, and then subsequently incubated with primary antibodies against human Aβ (6E10, BioLegend, 803014, 1:400) and LAMP1 (Abcam, ab24170, 1:200) overnight at 4°C, and appropriate fluorescence-conjugated secondary antibodies (Thermo Fisher Scientific, A-11008, A-11005 or A-31577, 1:500) for 1 h at room temperature in the dark. Confocal images were acquired using the A1R (Nikon) or FV1000MPE-B (Olympus) confocal microscope. All images were processed with ImageJ to calculate the area of Aβ plaques and dystrophic neurites.

### Aβ ELISA Assays

Hippocampal tissues of treated 5xFAD mice were sequentially extracted with Tris-buffered saline (TBS), TBS containing 1% Triton X-100 (TBSX), and guanidine-HCl (GuHCl) supplemented with the Complete Protease Inhibitor Cocktail (Roche) as described previously (Youmans et al., 2011). Aβ40 and Aβ42 levels were measured using Human Aβ40 and Aβ42 ELISA Kits (Thermo Fisher Scientific, KHB3481 for Aβ40 and KHB3441 for Aβ42), following the manufacturer’s instructions.

### Oxygen Consumption Rate Measurement

Oxygen Consumption Rate (OCR) was studied using the Seahorse XF Cell Mito Stress Test Kit (Agilent, Santa Clara, CA, United States), with measurement on the Seahorse XFe 96 Extracellular Flux Analyzer (Agilent). Briefly, primary neurons from mice at embryonic day 16.5 (E16.5) were isolated and cultured for 7–10 days. 8–10 × 10^4^ neurons per well were plated on a Seahorse XF 96 cell culture microplate. After baseline detection, 1 μM oligomycin, 1.5 μM FCCP, and 1 μM rotenone-antimycin A were injected sequentially into the assay micro-chambers. Data were analyzed using the Seahorse Wave 2.2.0 software package (Agilent).

### Statistical Analysis

Statistical analysis was performed using GraphPad Prism 8 software. Data in figures were presented as mean ± SEM. Comparison of the mean values for multiple groups was performed using a one-way ANOVA or two-way ANOVA. Comparison of two groups was performed using unpaired *t*-test or Mann Whitney test. Exact sample sizes and statistical test used for each comparison were provided in corresponding figure legend. *p* < 0.05 was considered to be statistically significant.

## Results

### TMEM59 Overexpression Causes Memory Deficits in Mice

**FIGURE 1:**
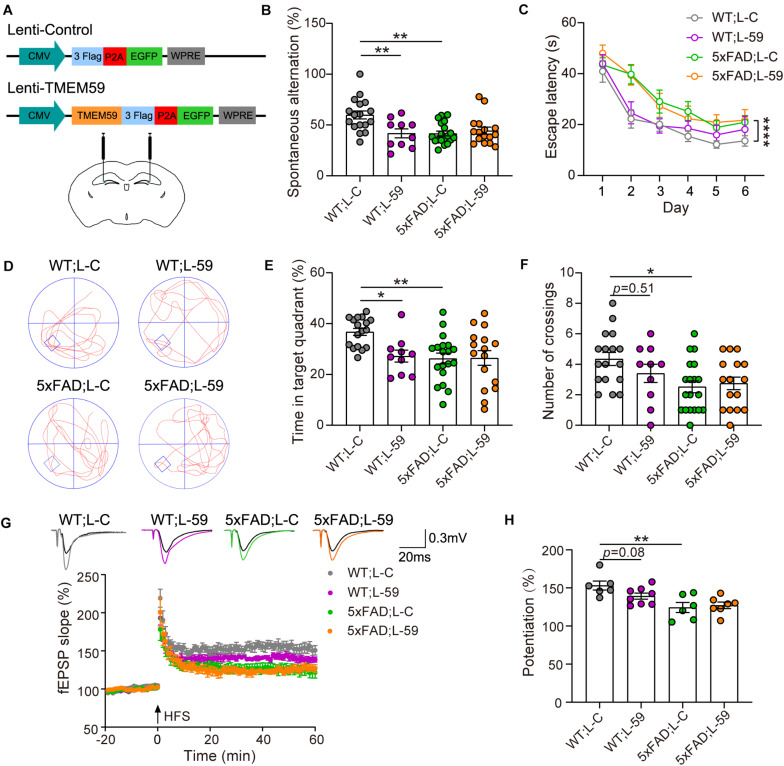
Overexpression of TMEM59 causes memory deficits in mice. **(A)** The schematic diagram of lentivirus constructs expressing TMEM59 (Lenti-TMEM59, L-59) or control (Lenti-Control, L-C) (upper panels), and their stereotactic injection into the hippocampal region (lower panel). **(B)** In Y maze tests, spontaneous alternation percentage of WT; L–C (*n* = 17), WT; L-59 (*n* = 10), 5xFAD; L-C (*n* = 19), and 5xFAD; L-59 (*n* = 16) mice were calculated for comparison. One-way ANOVA followed by Tukey’s *post hoc* test. **(C–F)** In Morris water maze tests, escape latencies during a 6-day training were recorded **(C)**. Representative swimming paths **(D)**, time spent in the target quadrant **(E)** and numbers of platform region crossings **(F)** during the probe test were also recorded. Comparisons were carried out for WT; L-C (*n* = 17), WT; L-59 (*n* = 10), 5xFAD; L-C (*n* = 19), and 5xFAD; L-59 (*n* = 16) mice. Two-way ANOVA followed by Tukey’s *post hoc* test for comparisons in **(C)**, and one-way ANOVA followed by Tukey’s *post hoc* test for comparisons in **(E,F)**. **(G)** Time course of fEPSP slopes in the hippocampal CA1 region in acute slices from WT; L-C, WT; L-59, 5xFAD; L-C, and 5xFAD; L-59 mice were recorded. **(H)** Quantifications and comparisons of average synaptic potentiation from the last 10 min shown in **(G)**. *n* = 6 slices for WT; L-C, *n* = 8 slices for WT; L-59, *n* = 6 slices for 5xFAD; L-C, and *n* = 7 slices for 5xFAD; L-59 from 4 to 5 mice per group, Mann Whitney test. Data represent mean ± SEM. *p < 0.05, **p < 0.01, ****p < 0.0001.

To ascertain whether TMEM59 dysregulation influences AD pathology, we delivered lentivirus expressing either TMEM59 (Lenti-TMEM59, tagged with Flag) or EGFP as a control (Lenti-Control) into the mouse hippocampus bilaterally at 2 months of age (Figure 1A). Exogenous TMEM59 expression was confirmed in these mice at about 8 months of age, and had no significant effect on endogenous TMEM59 levels (Supplementary Figures 2A,B). At 6–7 months of age, we found that overexpression of TMEM59 in WT and 5xFAD mice did not affect their total moving distance and time spent in the center during open field tests, suggesting that TMEM59 overexpression has no effect on mouse locomotor activity and anxiety (Supplementary Figures 2G,H). It has been reported that 5xFAD mice display reduced anxiety only at 9–12 months of age in open field tests (Jawhar et al., 2012). Here our results also confirmed that 5xFAD mice had no anxiety change at 6–7 months of age.

As expected, 6–7 month-old 5xFAD mice showed short-term working memory deficits compared to WT mice in *Y* maze tests (Figure 1B). Interestingly, overexpression of TMEM59 impaired short-term working memory in WT mice without further deteriorating the deficits in 5xFAD mice (Figure 1B). In Morris water maze tests, 5xFAD mice showed impaired spatial learning and memory with decreased escape latency during the training and reduced time spent in the target quadrant and numbers of platform region crossings during the probe test compared to WT controls (Figures 1C–F). While TMEM59 overexpression had no effect on altering these behaviors in 5xFAD mice, its overexpression in WT mice significantly reduced time spent in the target quadrant during the probe test (Figure 1E).

We next recorded LTP to test the effect of TMEM59 overexpression on synaptic plasticity (Figure 1G). We found that LTP was impaired in 5xFAD mice compared to WT controls. Moreover, TMEM59 overexpression moderately reduced LTP in WT mice without further compromising LTP deficits in 5xFAD mice (Figure 1H). Together, these results suggest that overexpression of TMEM59 leads to memory and synaptic plasticity impairments.

### TMEM59 Overexpression Exacerbates Aβ Deposition and Neurite Dystrophy in 5xFAD Mice

**FIGURE 2:**
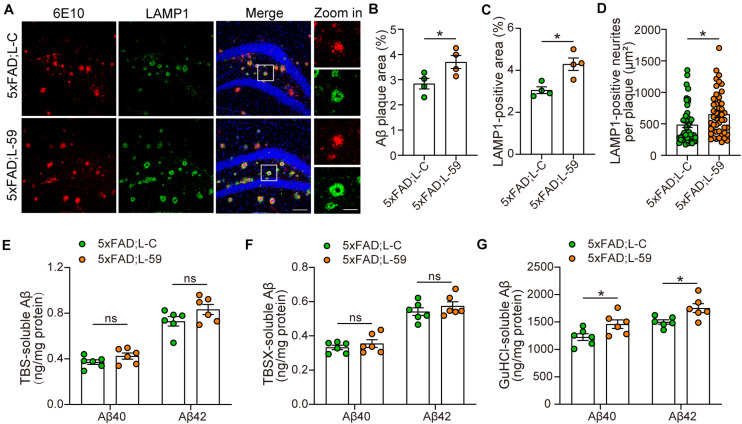
Overexpression of TMEM59 exacerbates Aβ plaque deposition and neurite dystrophy in 5xFAD mice. **(A)** Z-stack confocal images of Aβ plaques (in red) and dystrophic neurites (indicated by LAMP1, in green) in the coronal sections from 6 to 7 month-old 5xFAD; L-C and 5xFAD; L-59 mice. Original magnifications are 20×, scale bar, 100 μm. Zoom-in images are on the right, scale bar, 30 μm. **(B,C)** Quantifications and comparisons of the total area of Aβ plaques **(B)** and LAMP1-positive dystrophic neurites **(C)** shown in **(A)**. *n* = 4 mice per group. **(D)** Quantifications and comparisons of the area of Aβ-associated LAMP1-positive dystrophic neurites per plaque shown in **(A)**. 48 plaques from four 5xFAD; L-C mice and 47 plaques from four 5xFAD; L-59 mice were studied for analysis. **(E–G)** The levels of Aβ40 and Aβ42 in the hippocampus of TBS-extractions **(E)**, TBSX-extractions **(F)**, and GuHCl-extractions **(G)** from 8 month-old 5xFAD; L-C and 5xFAD; L-59 mice were measured by ELISA and compared. *n* = 6 mice per group. Data represent mean ± SEM. *p < 0.05, ns: not significant. Unpaired *t*-test.

We also studied the impact of TMEM59 overexpression on Aβ plaque formation in 5xFAD mice. Immunofluorescence staining revealed that total Aβ plaque areas were dramatically increased in TMEM59-overexpressing 5xFAD mice compared to controls (Figures 2A,B). Total LAMP1-positive areas indicative of dystrophic neurites, as well as LAMP1-positive areas around each Aβ plaque were also markedly increased upon TMEM59 overexpression (Figures 2A,C,D). To further determine the effect of TMEM59 overexpression on Aβ, we carried out ELISA to measure Aβ levels in TBS-soluble, TBSX-soluble, and GuHCl-soluble extractions from mouse hippocampus, of which the formal two represent soluble or newly generated Aβ and the latter one represents detergent-insoluble deposited Aβ (Youmans et al., 2011). TMEM59 overexpression had no effect on Aβ40 and Aβ42 levels in TBS- and TBSX-soluble extractions (Figures 2E,F). However, Aβ40 and Aβ42 levels in the GuHCl-soluble fractions were significantly higher in TMEM59-overexpressing 5xFAD mice than in controls (Figure 2G). These findings reveal that TMEM59 upregulation exacerbates Aβ plaque deposition and neurite dystrophy in AD. One previous study showed that TMEM59 overexpression reduced APP glycosylation and Aβ generation (Ullrich et al., 2010). However, here we found that TMEM59 overexpression in the mouse hippocampus had no effect on levels of total APP, glycosylated APP, and APP processed α- and β-carboxyl-terminal fragment (CTF) (Supplementary Figures 2A,C–F).

### TMEM59 Haploinsufficiency Reverses Memory and Synaptic Plasticity Deficits in 5xFAD Mice

**FIGURE 3:**
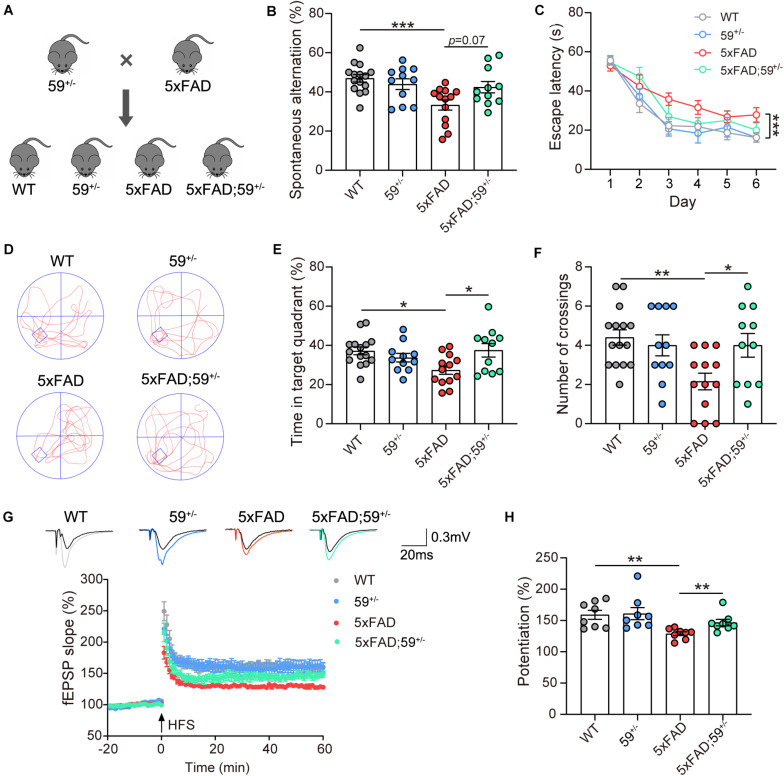
TMEM59 haploinsufficiency attenuates memory and synaptic plasticity deficits in 5xFAD mice. **(A)** Schematic diagram for generating 5xFAD mice with TMEM59 haploinsufficiency. **(B)** In Y maze tests, spontaneous alternation percentage of WT (*n* = 15), 59^+/–^ (*n* = 11), 5xFAD (*n* = 13), and 5xFAD; 59^+/–^ (*n* = 11) mice were calculated for comparison. One-way ANOVA followed by Tukey’s *post hoc* test. **(C–F)** In Morris water maze tests, escape latencies during a 6-day training were recorded **(C)**. Representative swimming paths **(D)**, time spent in the target quadrant **(E)** and numbers of platform region crossings **(F)** during the probe test were also recorded. Comparisons were carried out for WT (*n* = 15), 59^+/–^ (*n* = 11), 5xFAD (*n* = 13), and 5xFAD; 59^+/–^ (*n* = 11) mice. Two-way ANOVA followed by Tukey’s *post hoc* test for comparisons in **(C)**, and one-way ANOVA followed by Tukey’s *post hoc* test for comparisons in **(E,F)**. **(G)** Time course of fEPSP slopes in the hippocampal CA1 region in acute slices from WT, 59^+/–^, 5xFAD, and 5xFAD; 59^+/–^ mice were recorded. **(H)** Quantifications and comparisons of average synaptic potentiation from the last 10 min shown in **(G)**. *n* = 8 slices from 4 to 5 mice per group, Mann Whitney test. Data represent mean ± SEM. *p < 0.05, **p < 0.01, ***p < 0.001.

To further evaluate the involvement of TMEM59 in AD, we first generated *Tmem59* conditional knockout (*Tmem59*^flox/flox^) mice. By crossing them with Zp3-Cre mice, we obtained *Tmem59* knockout (*Tmem59*^–/–^) mice (Supplementary Figures 1A–C). Crossing heterozygous *Tmem59* knockout (*Tmem59*^+/–^) mice with 5xFAD mice resulted in the generation of WT, *Tmem*59^+/–^ (59^+/–^), 5xFAD, and 5xFAD; *Tmem59*^+/–^ (5xFAD; 59^+/–^) mice (Figure 3A). As expected, 6–7 month-old 59^+/–^ and 5xFAD; 59^+/–^ mice showed reduced TMEM59 protein levels in the hippocampus compared to respective controls (Supplementary Figures 3A–B). The time spent in the center and total moving distance in open field tests of 59^+/–^ and 5xFAD; 59^+/–^ mice were comparable to respective controls (Supplementary Figures 3G,H), suggesting that TMEM59 haploinsufficiency did not affect mouse locomotor activity and anxiety at this age. However, TMEM59 haploinsufficiency had a moderate effect on rescuing short-term working memory deficits in 5xFAD mice in *Y* maze tests (Figure 3B). In Morris water maze tests, TMEM59 haploinsufficiency in 5xFAD mice significantly increased their time spent in the target quadrant and numbers of platform crossings during the probe test (Figures 3C–F). Moreover, TMEM59 haploinsufficiency rescued impaired LTP in 5xFAD mice (Figures 3G,H). Together, these results suggest that TMEM59 haploinsufficiency can ameliorate memory and synaptic plasticity deficits in 5xFAD mice.

### TMEM59 Haploinsufficiency Reduces Aβ Plaque Deposition and Neurite Dystrophy in 5xFAD Mice

**FIGURE 4:**
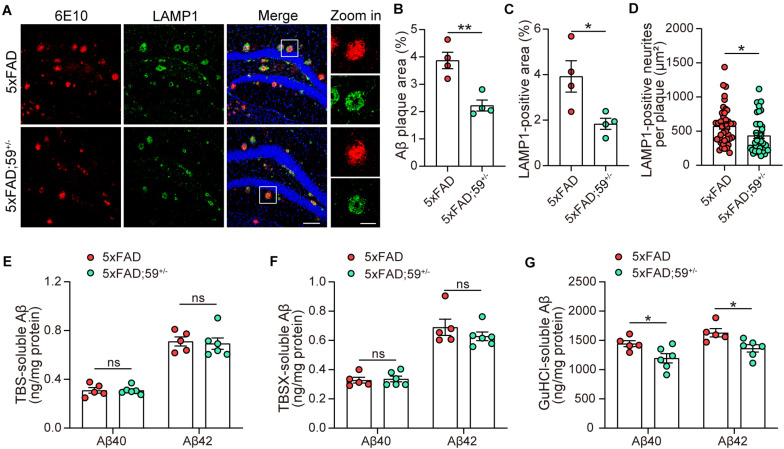
TMEM59 haploinsufficiency reduces Aβ plaque deposition and neurite dystrophy in 5xFAD mice. **(A)** Z-stack confocal images of Aβ plaques (in red) and dystrophic neurites (indicated by LAMP1, in green) in the coronal sections from 6 to 7 month-old 5xFAD and 5xFAD; 59^+/–^ mice. Original magnifications are 20×, scale bar, 100 μm. Zoom-in images are on the right, scale bar, 30 μm. **(B,C)** Quantifications and comparisons of the total area of Aβ plaques **(B)** and LAMP1-positive dystrophic neurites **(C)** shown in **(A)**. *n* = 4 mice per group. **(D)** Quantifications and comparisons of the area of Aβ-associated LAMP1-positive dystrophic neurites per plaque shown in **(A)**. 43 plaques from four 5xFAD mice and 40 plaques from four 5xFAD; 59^+/–^ mice were studied for analysis. **(E–G)** The levels of Aβ40 and Aβ42 in the hippocampus of TBS-extractions **(E)**, TBSX-extractions **(F)**, and GuHCl-extractions **(G)** from 8 month-old 5xFAD and 5xFAD; 59^+/–^ mice were measured by ELISA and compared. *n* = 5 mice for 5xFAD, *n* = 6 mice for 5xFAD; 59^+/–^. Data represent mean ± SEM. *p < 0.05, **p < 0.01, ns: not significant. Unpaired *t*-test.

We next studied the impact of TMEM59 haploinsufficiency on Aβ plaques in 5xFAD mice and found that Aβ plaque areas were reduced in 5xFAD; 59^+/–^ mice compared to 5xFAD mice (Figures 4A,B). Total dystrophic neurites and dystrophic neurites surrounding individual Aβ plaque, indicated by LAMP1-positive staining, were also decreased in 5xFAD; 59^+/–^ mice compared to controls (Figures 4A,C,D). We further measured Aβ levels by ELISA in sequential hippocampal extractions. Although not altered in TBS- and TBSX-soluble fractions, Aβ40 and Aβ42 levels were significantly decreased in GuHCl-soluble extractions in 5xFAD; 59^+/–^ mice compared to controls (Figures 4E–G). These results suggest that TMEM59 haploinsufficiency reduces Aβ plaque deposition and dystrophic neurite accumulation in 5xFAD mice. We also explored APP glycosylation and processing in mice with TMEM59 haploinsufficiency. The results showed that glycosylated APP levels were slightly increased in 5xFAD; 59^+/–^ mice compared to controls, whereas total APP and APP α-/β-CTF levels were not altered with TMEM59 haploinsufficiency (Supplementary Figures 3A,C–F).

### TMEM59 Protein Levels Are Elevated in the Brain of 5xFAD Mice During Aging

**FIGURE 5:**
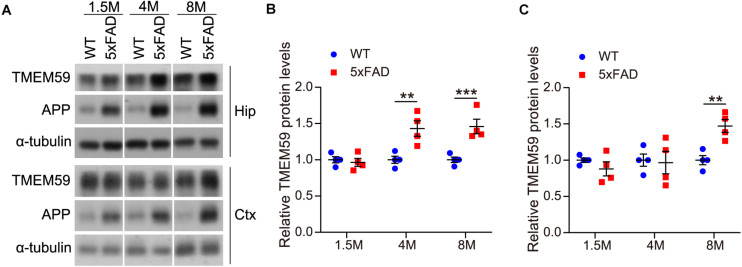
TMEM59 protein levels are increased in 5xFAD mice during aging. **(A)** Protein lysates of hippocampal (Hip) and cortical (Ctx) tissues from WT and 5xFAD mice at different ages were subjected to western blotting to detect proteins indicated. **(B,C)** Hippocampal **(B)** and cortical **(C)** TMEM59 protein levels in **(A)** were quantified and compared to respective controls (set to 1.0). Data represent mean ± SEM, *n* = 4 mice per group. **p < 0.01, ***p < 0.001. Two-way ANOVA followed by Bonferroni’s *post hoc* test.

Some previous studies reported that TMEM59 expression was increased in the brain of AD patients compared to controls (Bakulski et al., 2012; Guttula et al., 2012). Herein, we also observed that TMEM59 protein levels were elevated in 6–7 month-old 5xFAD mice compared to WT controls (Supplementary Figures 3A,B). To further determine the change of TMEM59 in AD, we studied TMEM59 levels in 5xFAD mice at different ages. We found that although hippocampal TMEM59 protein levels were comparable between 5xFAD mice and their littermate WT controls at 1.5 months of age, hippocampal TMEM59 levels were significantly elevated in 5xFAD mice compared to WT controls at 4 and 8 months of age (Figures 5A,B). Similarly, although TMEM59 protein levels in the cortex of 5xFAD mice were not altered at 1.5 and 4 months of age, they were significantly elevated at 8 months of age compared to WT controls (Figures 5A,C). These results indicate a correlation between TMEM59 elevation and AD and aging, and further support a detrimental role of TMEM59 elevation in synaptic functions and learning and memory.

## Discussion

TMEM59 has been implicated to play a role in AD (Ullrich et al., 2010; Bakulski et al., 2012; Guttula et al., 2012). However, whether TMEM59 indeed modulates the pathology of AD, especially in animal models resembling AD phenotypes has yet to ascertained. In the present study, we observed that TMEM59 protein levels were significantly elevated in 5xFAD mice during aging; and this is consistent with the previous reports of high *TMEM59* expression levels and low DNA methylation in the *TMEM59* promoter region in AD patients compared to controls (Bakulski et al., 2012; Guttula et al., 2012), strengthening the correlation between TMEM59 and AD.

Cognitive impairment is regarded as a typical feature of AD in clinical diagnosis (McKhann et al., 2011). Herein, we found that lentivirus-mediated TMEM59 overexpression in the hippocampal region was sufficient to cause memory deficits and had a trend to impair synaptic plasticity in WT mice, implying that increased TMEM59 expression contributes to AD progression. However, TMEM59 overexpression did not exacerbate learning and memory and synaptic plasticity deficits in 5xFAD mice. One possible explanation is that the impacts of TMEM59 overexpression on cognitive and synaptic function impairments are mild and may not be able to further worsen the quickly degenerated phenotypes in 5xFAD mice. It is well-known that 5xFAD mice develop AD-like phenotypes very fast and such an aggressive phenotype in some ways is unphysiological to the human disease. Further study using models with relatively slow disease progression such as APP/PS1 and Tg2576 mice may be able to fully ascertain the contribution of TMEM59 elevation to AD progression.

Aβ is considered to be a prime culprit for AD pathogenesis and derived from APP through sequential cleavages by β-secretase and γ-secretase (Hardy, 2006; Zhang et al., 2011; Haass et al., 2012; Long and Holtzman, 2019). Aβ40 and Aβ42 are two major neurotoxic species among various Aβ species, with Aβ42 being more prone to aggregate into oligomers, fibrils and amyloid plaques in AD patients (Jarrett et al., 1993; Iwatsubo et al., 1994; Tu et al., 2014; Long and Holtzman, 2019). Interestingly, we found that TMEM59 overexpression exacerbated Aβ deposition in the brain of 5xFAD mice. We also studied Aβ40 and Aβ42 levels in 5xFAD mouse hippocampal fractions after sequential extraction by TBS, TBSX, and GuHCl, of which the formal two represent soluble or newly generated Aβ and the latter one represents detergent-insoluble deposited Aβ (Youmans et al., 2011; Zhong et al., 2019). Consistently, both Aβ40 and Aβ42 levels in GuHCl-soluble fractions were increased upon TMEM59 overexpression. However, TMEM59 overexpression had no effect on Aβ40 and Aβ42 levels in TBS- and TBSX-soluble fractions, implying that TMEM59 overexpression may not affect Aβ generation. This finding is in contrast to previous studies showing that TMEM59 overexpression could inhibit APP glycosylation and cell surface expression, as well as the cleavage of APP to generate Aβ in HEK293 cells (Ullrich et al., 2010), and that TMEM59 overexpression could reduce the γ-cleavage of APP C99 fragment and promote learning and memory in C99 transgenic drosophila (Li et al., 2020). Although our finding that APP glycosylation was increased in 5xFAD mice with TMEM59 haploinsufficiency is consistent with the observation that TMEM59 overexpression inhibited APP glycosylation (Ullrich et al., 2010), TMEM59 haploinsufficency had no effect on levels of total APP, APP α-/β-CTF, and soluble Aβ *in vivo*. One possibility for this discrepancy is that TMEM59 may have different effects on APP/Aβ metabolism in immortalized non-neuronal cell lines and animal models.

The formation of dystrophic neurites is also one pathological trait in AD (Holcomb et al., 1998; Guo et al., 2020). Many previous studies have demonstrated that LAMP1, a lysosomal marker for endo-lysosomal and autophagic vesicles, is enriched in dystrophic neurites and accumulates around Aβ plaques in AD mouse models (Condello et al., 2011; Gowrishankar et al., 2015; Yuan et al., 2016) as well as in AD patients (Terry et al., 1964; Barrachina et al., 2006; Hassiotis et al., 2018). Therefore, LAMP1 staining has been used as a marker for dystrophic neurites. Herein, we also found that TMEM59 overexpression resulted in increased staining of LAMP1, emphasizing the pathologic contribution of TMEM59 elevation on neurite dystrophy in AD.

One research group reported that complete knockout and nervous system-specific knockout of *Tmem59* resulted in memory impairments in mice (Liu et al., 2018; Wang et al., 2019). In contrast, we found that TMEM59 haploinsufficiency had no effect on learning and memory and synaptic plasticity in WT mice. Importantly, TMEM59 haploinsufficiency reverses memory and synaptic plasticity deficits in 5xFAD mice. Consistently, TMEM59 haploinsufficiency reduces Aβ deposition, detergent-insoluble but GuHCl-soluble Aβ42 levels, as well as dystrophic neurites in the brain of 5xFAD mice. Therefore, downregulation of TMEM59 can provide protection in AD. We recently demonstrated TMEM59 deficiency in microglia resulted in elevated phagocytosis and mitochondrial respiration (Liu et al., 2020). Herein, we also found the basal respiratory capacity of mitochondria was enhanced in primary neurons derived from TMEM59 knockout mice when compared to controls (Supplementary Figures 4A,B). Therefore, one potential mechanism for TMEM59 haploinsufficiency to exert protection in AD is that TMEM59 haploinsufficiency increases microglial phagocytosis of Aβ and promotes cellular health.

In summary, our study demonstrates that an elevation of TMEM59 can exacerbate the pathological progress during aging, whereas downregulation of TMEM59 can ameliorate cognitive and synaptic deficits and pathologies in AD model mice. These findings strongly support the notion that TMEM59 plays an important role in AD pathogenesis and may provide a potential strategy for AD treatment.

## Data Availability Statement

All datasets presented in this study are included in the article/Supplementary Material.

## Ethics Statement

The animal study was reviewed and approved by Animal Ethics Committee of Xiamen University.

## Author Contributions

JM and YZ designed the experiments. JM and LH performed most molecular and animal experiments. NZ carried out electrophysiological experiments under supervision by HS. HiX and ZL helped with animal experiments. XZ, HL, and DC provided technical supports. JM, LH, HaX, and YZ interpreted the data and wrote the manuscript. YZ supervised the project. All authors reviewed the manuscript.

## Conflict of Interest

The authors declare that the research was conducted in the absence of any commercial or financial relationships that could be construed as a potential conflict of interest.
