# Supplementary figures and images for: TMEM59 Haploinsufficiency Ameliorates the Pathology and Cognitive Impairment in the 5xFAD Mouse Model of Alzheimer’s Disease
Source: Front Cell Dev Biol. 2020 Oct 28;8:596030. doi: 10.3389/fcell.2020.596030 (PMC7655972; doi:10.3389/fcell.2020.596030)

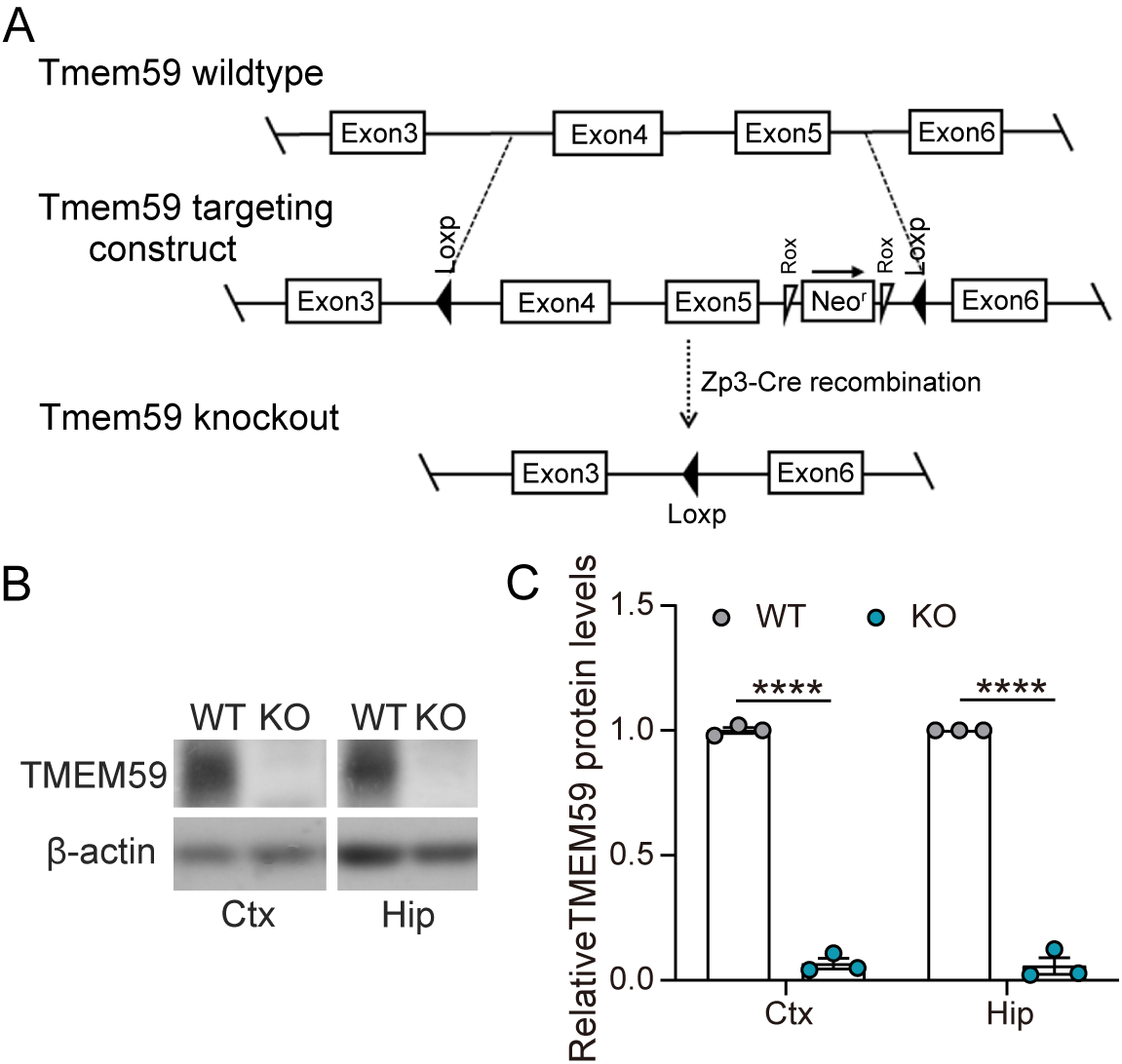

Supplement: Supplementary file 1 [file Image_1.TIF]

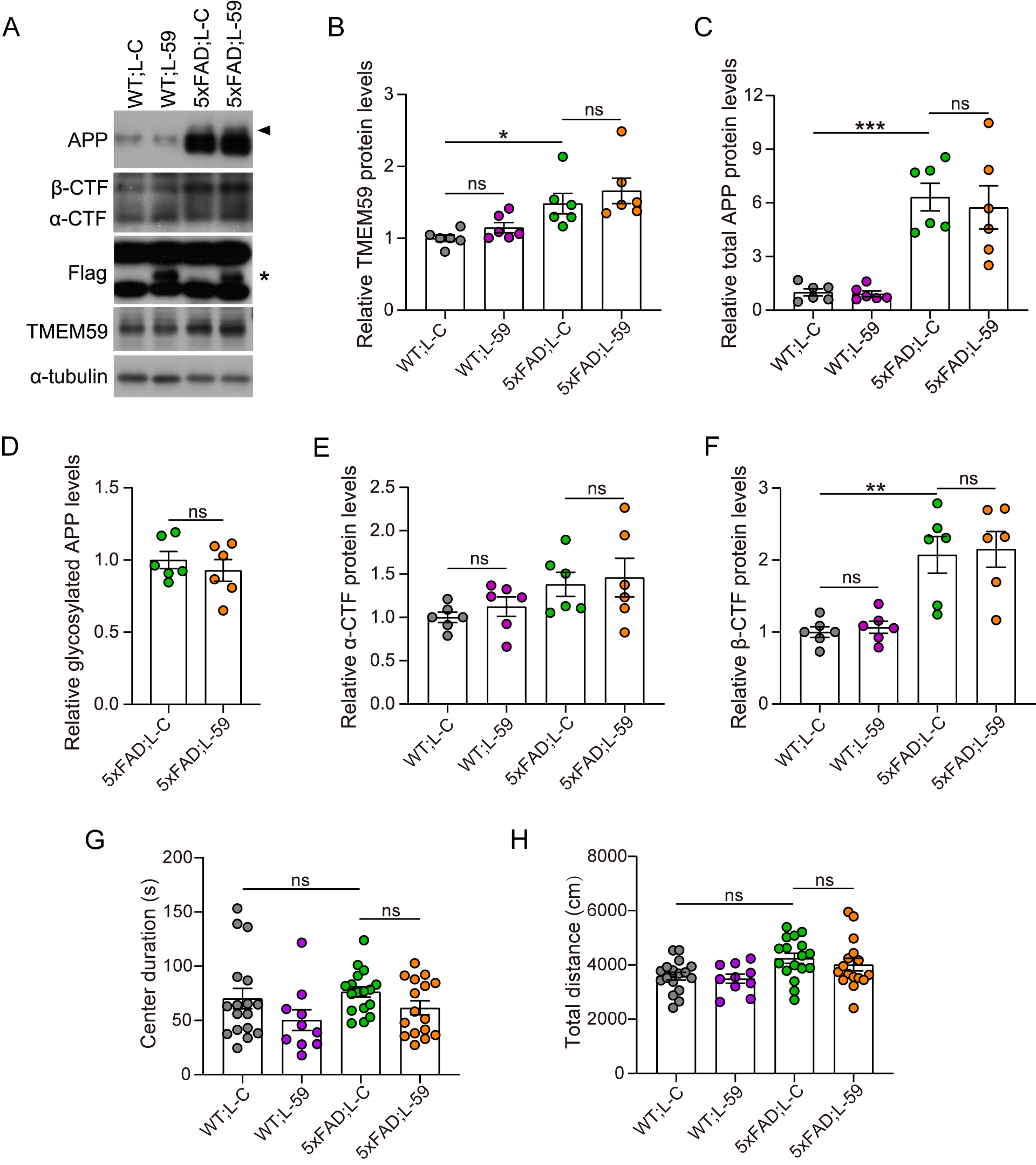

Supplement: Supplementary file 2 [file Image_2.TIF]

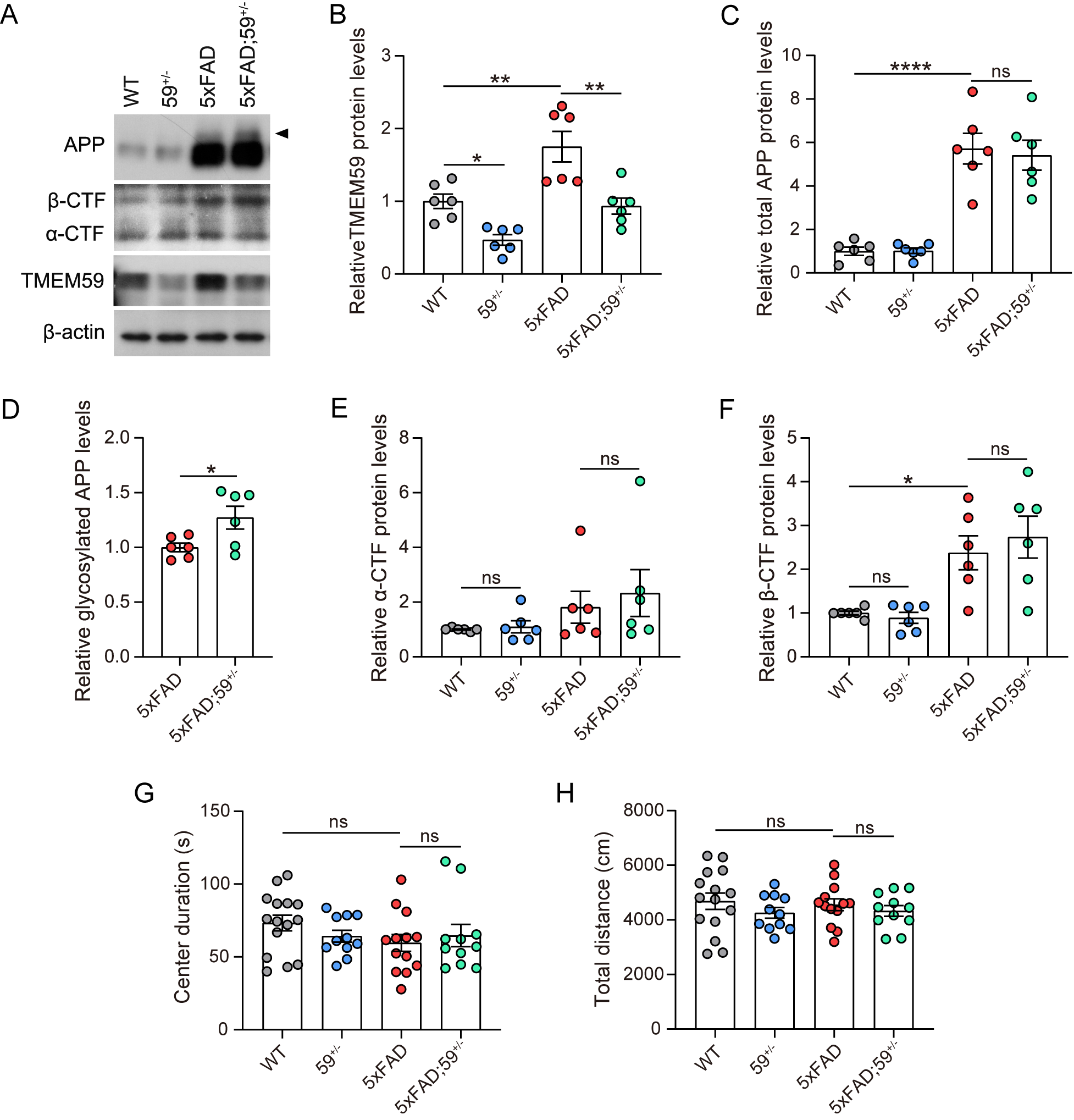

Supplement: Supplementary file 3 [file Image_3.TIF]

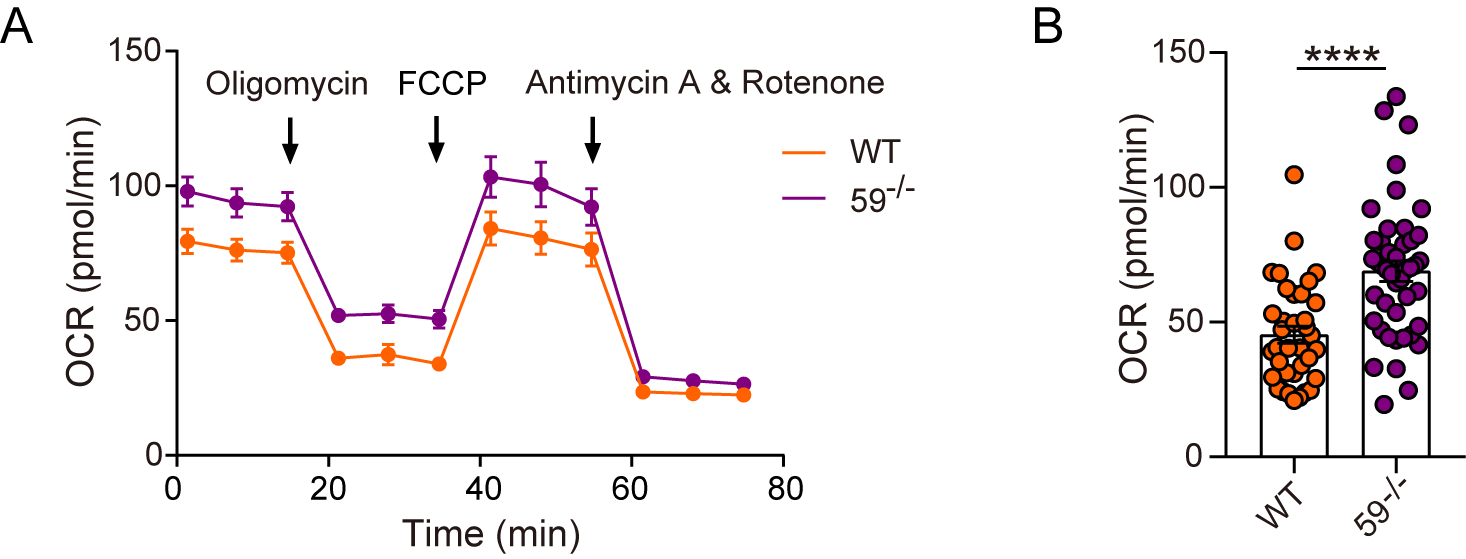

Supplement: Supplementary file 4 [file Image_4.TIF]

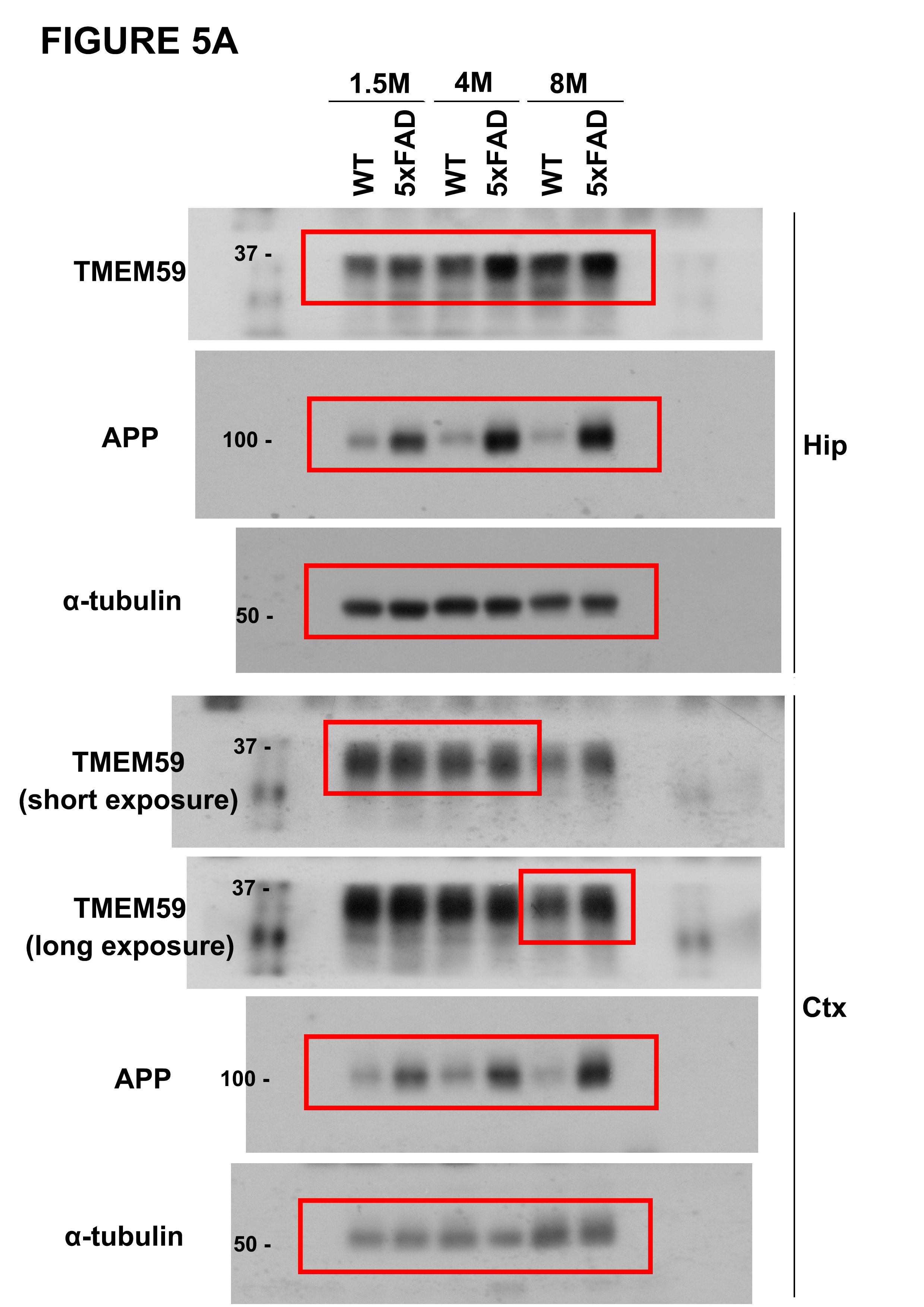

Supplement: Supplementary file 5 [file Data_Sheet_1.ZIP › Original data/Fig5A.jpg]

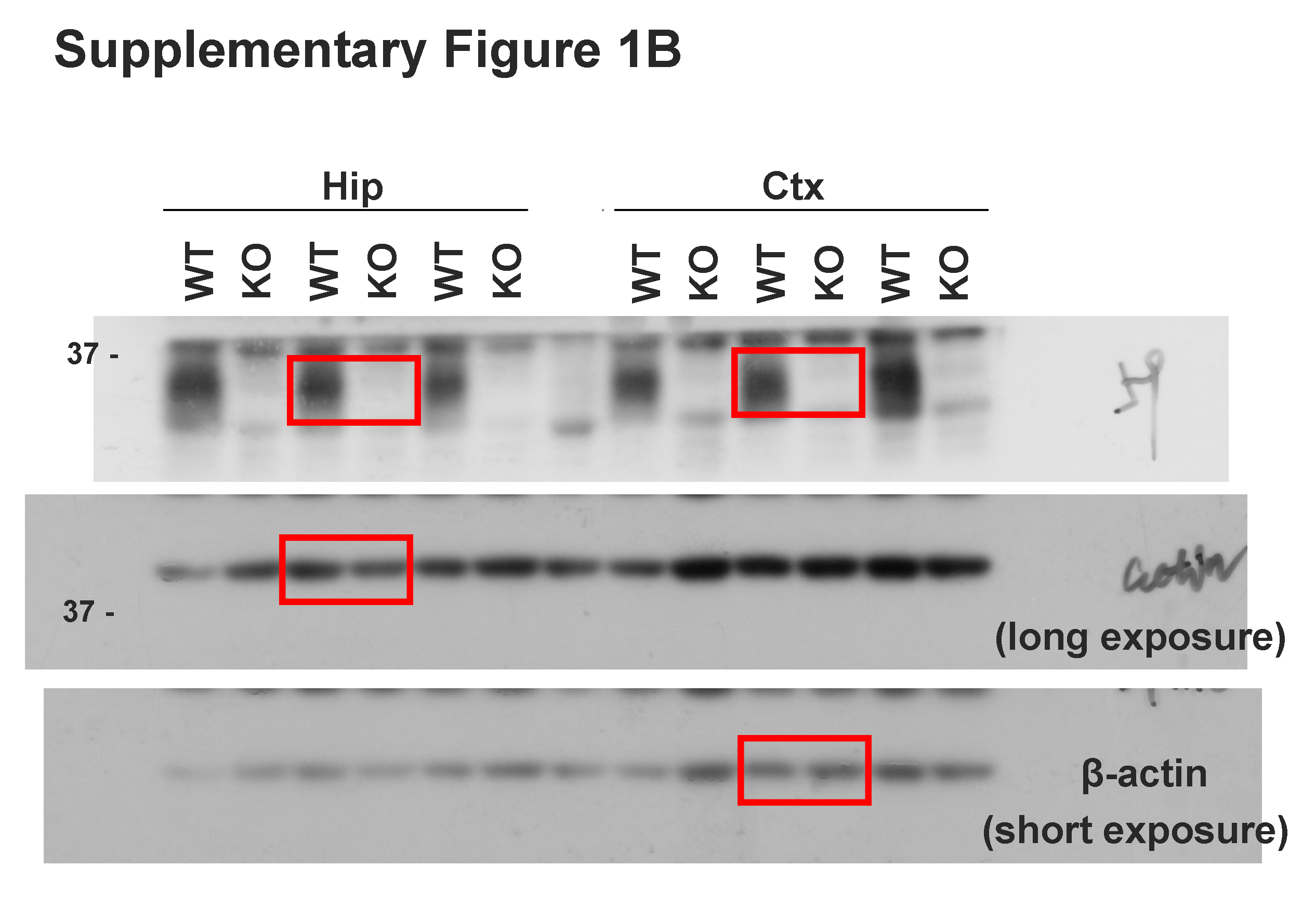

Supplement: Supplementary file 5 [file Data_Sheet_1.ZIP › Original data/Supplementary Figure 1B.jpg]

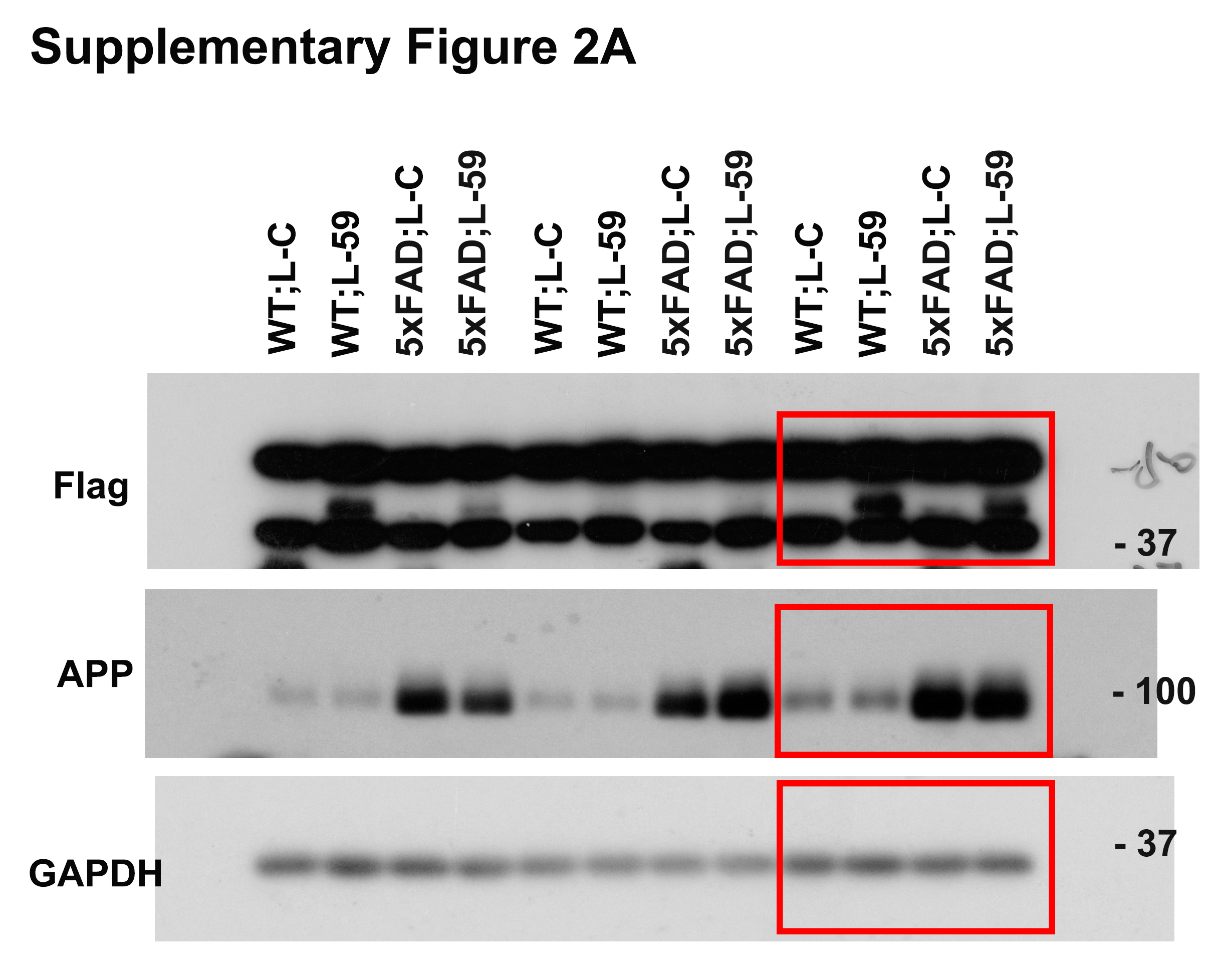

Supplement: Supplementary file 5 [file Data_Sheet_1.ZIP › Original data/Supplementary Figure 2A.jpg]

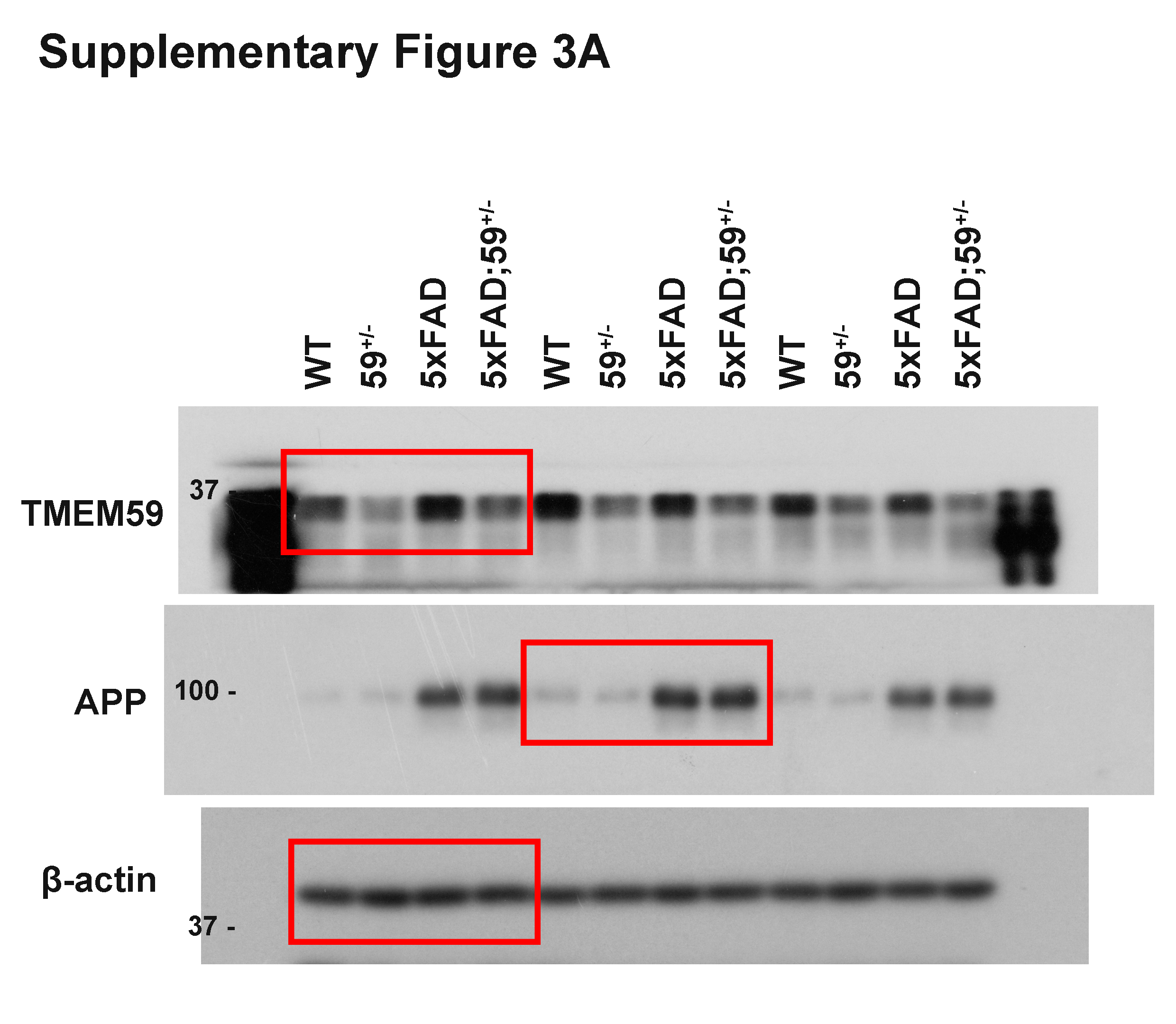

Supplement: Supplementary file 5 [file Data_Sheet_1.ZIP › Original data/Supplementary Figure 3A.jpg]
