# Supplementary material for: TMEM59 Haploinsufficiency Ameliorates the Pathology and Cognitive Impairment in the 5xFAD Mouse Model of Alzheimer’s Disease
Source: Front Cell Dev Biol. 2020 Oct 28;8:596030. doi: 10.3389/fcell.2020.596030 (PMC7655972; doi:10.3389/fcell.2020.596030)
Supplement: Supplementary file 6 [file Data_Sheet_2.PDF]

# TMEM59 Haploinsufficiency Ameliorates the Pathology and Cognitive Impairment in the 5xFAD Mouse Model of Alzheimer's Disease


Jian Meng[1†], Linkun Han[1†], Naizhen Zheng[1†], Hui Xu[1], Zhaoji Liu[1,2], Xian Zhang[1], Hong Luo[1], Dan Can[1], Hao Sun[1], Huaxi Xu[1], Yun-wu Zhang[1,3*]

[1]Fujian Provincial Key Laboratory of Neurodegenerative Disease and Aging Research, Institute of Neuroscience, School of Medicine, Xiamen University, Xiamen, Fujian 361102, China

[2]Department of Neurology, Zhongshan Hospital Xiamen University, Xiamen, Fujian 361004, China

[3]Department of Neurology, The First Affiliated Hospital of Xiamen University, Xiamen, Fujian 361003, China


## *Supplementary Materials*

## SUPPLEMENTARY FIGURE LEGENDS

**Supplementary Figure 1.** Generation of *Tmem59*$^{-/-}$ mice. **(A)** The strategy used for constructing *Tmem59* conditional knockout (cKO) mice (*Tmem59*$^{flox/flox}$). *Tmem59*$^{flox/flox}$ mice were crossed with Zp3-Cre mice to generate *Tmem59*$^{-/-}$ (knockout, KO) mice. **(B)** Western blotting showing the loss of TMEM59 protein in *Tmem59* KO mice compared to wild type (WT) mice. **(C)** TMEM59 protein levels in **(B)** were quantified and normalized to those of WT (set to 1.0) for comparison. n = 3 mice per group. Data represent mean ± SEM. ****$p < 0.0001$. Unpaired *t* test.

**Supplementary Figure 2.** Overexpression of TMEM59 has no effect on locomotor activity and APP processing in mice. **(A)** Protein lysates of hippocampal tissues from 6-7 month-old WT;L-C, WT;L-59, 5xFAD;L-C, and 5xFAD;L-59 mice were subjected to western blotting to detect proteins indicated. Glycosylated APP was indicated by an arrowhead. Flag-tagged exogenous TMEM59 was detected by an anti-Flag antibody and indicated by an asterisk. **(B-F)** TMEM59 **(B)**, total APP **(C)**, glycosylated APP **(D)**, APP α-CTF **(E)**, and APP β-CTF **(F)** protein levels were quantified and normalized to those of WT;L-C (set to 1.0) for comparison. n = 6 mice per group. Unpaired *t* test for two groups or one-way ANOVA followed by Tukey's post hoc test for multiple groups. (**G, H**) In open field tests, time spent in center arena (**G**) and total distance travelled (**H**) by WT;L-C (n = 17), WT;L-59 (n = 10), 5xFAD;L-C (n = 17), and 5xFAD;L-59 (n = 16) mice were recorded for comparison. One-way ANOVA followed by Tukey's post hoc test. Data represent mean ±

SEM. *$p < 0.05$, **$p < 0.01$, ***$p < 0.001$, ns: not significant.

**Supplementary Figure 3.** TMEM59 haploinsufficiency has no effect on locomotor activity and APP processing in mice. **(A)** Protein lysates of hippocampal tissues from 6-7 month-old WT, $59^{+/-}$, 5xFAD, and 5xFAD;$59^{+/-}$ mice were subjected to western blotting to detect proteins indicated. Glycosylated APP was indicated by an arrowhead. **(B-F)** TMEM59 **(B)**, total APP **(C)**, glycosylated APP **(D)**, APP α-CTF **(E)**, and APP β-CTF **(F)** protein levels were quantified and normalized to those of WT (set to 1.0) for comparison. n=6 mice per group. Unpaired *t* test for two groups or one-way ANOVA followed by Tukey's post hoc test for multiple groups. (**G, H**) In open field tests, time spent in center arena (**G**) and total distance travelled (**H**) by WT (n = 15), $59^{+/-}$ (n = 11), 5xFAD (n = 13), and 5xFAD;$59^{+/-}$ (n = 11) mice were recorded for comparison. One-way ANOVA followed by Tukey's post hoc test. Data represent mean ± SEM. *$p < 0.05$, **$p < 0.01$, ****$p < 0.0001$, ns: not significant.

**Supplementary Figure 4.** TMEM59 deficiency promotes the respiratory capacity of mitochondria in primary neurons. **(A)** Mouse primary neurons from Wild type (WT) and *Tmem59*$^{-/-}$ mice were cultured in assay micro-chambers. Oligomycin, FCCP, and Rotenone plus antimycin A were injected sequentially as indicated into the micro-chamber. Traces of the oxygen consumption rate (OCR) were recorded. **(B)** Quantifications and comparisons

of basal OCR shown in **(A)**. Data represent mean ± SEM, n = 36 for WT and n = 44 for *Tmem59*$^{-/-}$ from 3 independent experiments, ****$p < 0.0001$, unpaired *t* test.
